# A Rare Case of Human Coronavirus 229E Associated with Acute Respiratory Distress Syndrome in a Healthy Adult

**DOI:** 10.1155/2018/6796839

**Published:** 2018-04-15

**Authors:** Foula Vassilara, Aikaterini Spyridaki, George Pothitos, Athanassia Deliveliotou, Antonios Papadopoulos

**Affiliations:** ^1^Hygeia Hospital, Athens, Greece; ^2^4th Department of Internal Medicine, Medical School, National and Kapodistrian University of Athens, Athens, Greece

## Abstract

Human coronavirus 229E (HCoV-229E) is one of the first coronavirus strains being described. It is linked to common cold symptoms in healthy adults. Younger children and the elderly are considered vulnerable to developing lower respiratory tract infections (LRTIs). In particular, immunocompromised patients have been reported with severe and life-threatening LRTIs attributed to HCoV-229E. We report for the first time a case of LRTI and acute respiratory distress syndrome developed in a healthy adult with no comorbidities and HCoV-229E strain identified as the only causative agent. A 45-year-old female with a clear medical history presented with fever, cough, and headache. Respiratory tract infection was diagnosed, and empirical antibiotics were started. Within two days, she developed bilateral pleural effusions, diffuse consolidations, and ground glass opacities involving all lung fields. She needed immediate oxygen supply, while ABGs deteriorated and chest imaging and PaO_2_/FiO_2_ indicated ARDS. Early administration of systemic corticosteroids led to gradual clinical improvement. Multiplex PCR from nasal secretions was positive only for HCoV-229E and negative for multiple other pathogens. It remains to be elucidated how an immunocompetent adult developed a life-threatening LRTI caused by a “benign considered” coronavirus strain, the HCoV-229E.

## 1. Introduction

Coronaviruses (CoVs), a genus of the Coronaviridae family, are positive-stranded RNA viruses. The first human coronavirus (HCoV) appeared in reports in the mid-1960s and was isolated from persons with common cold. Two species were first detected: HCoV-229E and subsequently HCoV-OC43 [1, 2]. Since then, more species were described [3–5].

The HCoV-229E strain was associated with common cold symptoms [6]. Younger children and the elderly were considered more vulnerable to lower respiratory tract infections. Severe lower respiratory tract infection so far has only been described in immunocompromised patients [7, 8]. To our knowledge, there is no report describing life-threatening conditions in immunocompetent adults attributed to HCoV-229E. We report a case of acute respiratory distress syndrome developed in a healthy adult with no comorbidities and HCoV-229E strain identified as the only causative agent.

## 2. Case Presentation

A 45-year-old female patient presented to the emergency department with dry cough, headache, and fever up to 39.5°C lasting a few hours. Her past medical history was unremarkable, and she did not take any medication regularly. She has never smoked, worked as a teacher at a local high school, and has not recently travelled.

Clinical examination revealed rales at her left lower lung fields. Chest X-ray showed diffuse opacities and consolidation at this field. The arterial blood gases (ABGs) were normal, and intravenous ceftriaxone and azithromycin were empirically administered for lower respiratory tract infection (LRTI). *S. pneumoniae* and *L. pneumophila* antigen in the patient's urine specimen was negative, and blood cultures were sterile.

**Figure 1:**
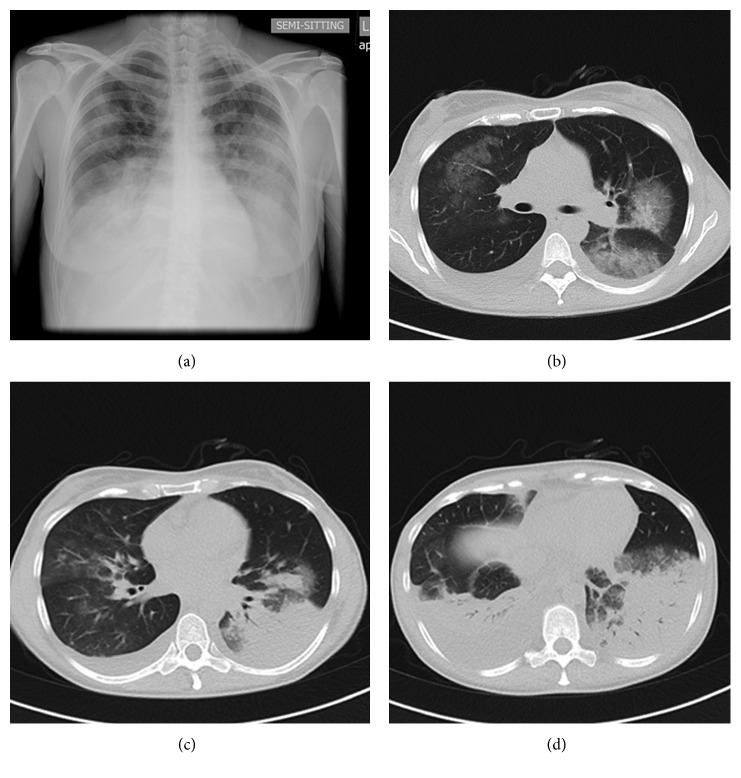
Chest CT scan and chest X-ray (semisitting position, posterior-anterior view) of the patient after clinical deterioration depicting diffuse bilateral opacities.

Over the next two days, the patient's clinical condition rapidly deteriorated, with development of tachypnea (34 respirations/minute), dyspnea, and hypoxemia. ABGs changed to PaO_2_ of 55.3 mmHg, PCO_2_ of 31.4 mmHg, and pH of 7.487. Lung auscultation revealed diffuse rhonchi symmetrically all over her chest, bronchial breathing at her right and left lower lobes, and diminished vesicular sounds. Chest CT scan displayed bibasilar pleural effusions and diffuse consolidations plus ground glass opacities involving all lung fields (Figure 1). Oxygen was supplied at 5 L/min, and antimicrobial therapy was changed to levofloxacin 500 mg/day. Systemic corticosteroids and bronchodilators were added about 40 hours after her hospitalization. Samples of the pleural fluid showed exudate with 260 cells/mm^3^, negative Gram stain, and sterile cultures.

Nasal secretions were collected, and multiplex PCR technology was applied targeting multiple pathogens (RespiFinder® 22, PathoFinder), including coronavirus 229E; coronavirus NL63, HKU1, and OC43; influenza A, B, and H1N1; parainfluenza 1, 2, 3, and 4; *Mycoplasma pneumoniae*; *Legionella pneumophila*; *Bordetella pertussis*; bocavirus; rhinovirus/*Enterovirus*; adenovirus; RSV A and B; and *Chlamydophila pneumoniae*. The result was positive for HCoV-229E, while negative for the other tested pathogens; PCR for SARS-CoV and MERS-CoV was also negative.

**Figure 2:**
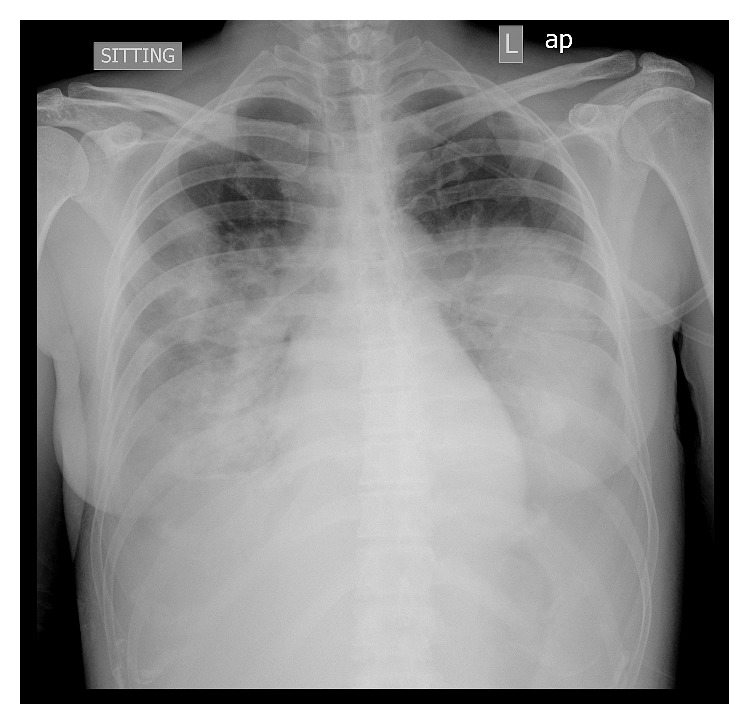
The patient's chest X-ray showing extensive bilateral airspace disease consistent with ARDS.

Within the next few hours, the patient's clinical condition further worsened and she required increased oxygen supply. New ABGs showed PaO_2_ = 76 mmHg, PCO_2_ = 33 mmHg, and pH = 7.45 at FiO_2_ = 0.50 with PaO_2_/FiO_2_ = 152, indicating ARDS. The patient was in severe respiratory distress and remained febrile and tachypneic, and a new chest X-ray showed multiple consolidations all over her lung fields (Figure 2). Intravenous linezolid was added to her regimen empirically in order to treat a possible community-acquired *Staphylococcus aureus* pneumonia.

**Figure 3:**
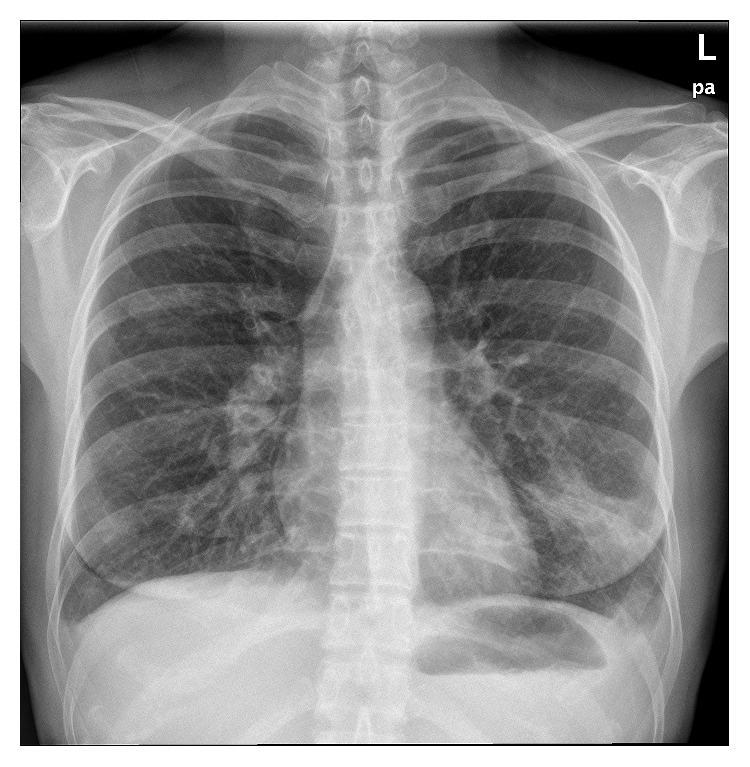
Chest X-ray at the patient's exit.

A repeat one-step RT-PCR in a nasal sample (Taqman, in-house protocol, Hellenic Pasteur Institute) confirmed the exclusive presence of human coronavirus 229E (HuCoV-229E). After the administration of systemic corticosteroids, the patient started to display clinical improvement within the first 24 hours. Further laboratory analyses did not reveal any immune defect. After a week, she was discharged from the hospital well and remained healthy 23 months later (Figure 3).

## 3. Discussion

The initially described coronavirus strain 229E has been previously identified as the second most frequent cause of common cold after rhinoviruses in healthy adults. Predominant symptoms were acute rhinorrhea, nasal congestion, and/or sore throat [9, 10]. Nasal discharge was the hallmark of all symptoms after inoculation of HuCoV-229E to healthy volunteers, and further observed symptoms were malaise, headache, chills, and cough [6].

HCoV-229E has been associated with bronchitis, acute exacerbations of COPD, and pneumonia in infants, children, and elderly persons with underlying illnesses [11–13]. Life-threatening infections have only been described in immunocompromised patients [7, 8], but the correlation of HCoV-229E with LRTI in healthy adult individuals is uncertain [9]. An adult patient with pneumonia tested positive for HCoV-229E has been described in a study conducted in rural Thailand, but it is not made clear if other comorbidities were present [14]. Nine Italian patients hospitalized with LRTI have also been tested positive for HCoV-229E; however, their age is not specified [15]. Αlthough numerous studies have tentatively linked 229E infections to severe respiratory tract illness over many years, no study controlling for age and underlying illness has demonstrated an epidemiologic association between infection with HcoV-229E in healthy adults and any illness other than the common cold. Furthermore, no case of HCoV-229E-associated ARDS has been reported in immunocompetent adults. Only a few cases of pulmonary infection and ARDS have been described in a 76-year-old woman infected with the closely related alpha coronavirus HCoV-NL63 [16] and in a 39-year-old woman with poorly controlled DM and infected with the beta coronavirus HCoV-OC43.

The patient was a teacher and thus exposed to multiple pathogens from her students. She was an immunocompetent adult with no underlying disease. Her symptoms progressed rapidly, despite the immediate administration of broad-spectrum antibiotics, and clinical, laboratory, and radiologic findings were compatible with ARDS [17]. The patient came very close to intubation and mechanical ventilation, but early addition of corticosteroids in her therapeutic regimen seems to have played a decisive role towards her favorable outcome. Close monitoring and continuous recording and assessment of her vital signs warranted the borderline avoidance of her transfer to the ICU.

HCoV-229E was isolated twice from the patient's nasal secretions; she was not intubated, and thus, the BAL sample was not taken. Extensive workup did not reveal any immune defect; all microbiological and serological studies remained negative for other pathogens. Rapid and reliable diagnosis of human coronavirus infections is of pronounced clinical importance. New RT-PCR methods [18] in sputum and nasal aspirates successfully have diagnosed human coronavirus infections. Multiplex RT-PCR is used increasingly to diagnose respiratory infections and has shown to be more sensitive than viral culture and antigen detection and also rapid and cost-effective [19], with greater sensitivity and similar specificity compared to real-time RT-PCR [20].

## 4. Conclusion

To our knowledge, it is the first time that human coronavirus HCoV-229E has been detected in severe lower respiratory tract infection with ARDS of a healthy adult with no comorbidities. Although it is considered as a “benign” microorganism and linked to mild respiratory symptoms, the presence of HCoV-229E should not be underestimated and considered as a possible pathogen even in coinfections with other microorganisms and in more serious LRTIs. The reason why HuCoV-229E causes different clinical manifestations in diverse patient groups has not yet been answered. The process through which HCoV-229E may evade normal immune defense and cause life-threatening illness remains to be elucidated.
